# Is regulatory science ready for artificial intelligence?

**DOI:** 10.1038/s41746-025-01596-0

**Published:** 2025-04-10

**Authors:** Thomas Hartung, Maurice Whelan, Weida Tong, Robert M. Califf

**Affiliations:** 1 Johns Hopkins University, Bloomberg School of Public Health and Whiting School of Engineering, Baltimore, MD USA; 2 Department of Biology, University of Konstanz, Konstanz, Germany; 3 European Commission, Joint Research Center (JRC), Ispra, Italy; 4 National Center for Toxicological Research, US Food and Drug Administration, Jefferson, AR USA; 5 Office of Commissioner, US Food and Drug Administration, Silver Spring, MD USA

**Keywords:** Government, Computer science, Computational biology and bioinformatics, Public health

## Abstract

Trust is key in AI for regulatory science, but its definition is debated. If AI models use different features yet perform similarly, which should be trusted? If scientific theories must be testable, how critical is explainability? At the Global Summit on Regulatory Science (GSRS24), regulators agreed that successful AI adoption requires ongoing dialogue, adaptability, and AI-trained personnel to harness its potential for regulatory responsibilities in the evolving 21st-century landscape.

## Introduction

The Global Coalition for Regulatory Science Research (GCRSR), established under the leadership of former FDA Commissioner Dr. Margaret A. Hamburg, aims at engaging global regulators to advance regulatory science by “bringing 21st century approaches to 21st century products and problems”^1^. Today, GCRSR comprises 16 regulatory agencies from 11 countries and the European Union, and its mission focuses on adopting emerging technologies and big data science to improve regulatory science research on the safety and efficacy of foods and drugs^2^. One of its main activities is the annual Global Summit on Regulatory Science (GSRS) conference, which brings together regulators, industry, and academia to discuss emerging technologies and New Approach Methods that support future regulatory application. This year, the 14th GSRS was hosted by FDA with a theme on Digital Transformation in Regulatory Science^3^. This 3-day event with speakers from across the globe, including the United States, Brazil, Canada, China, Germany, Italy, Japan, Saudi Arabia, Singapore, Switzerland, and the United Kingdom. The main program included seven sessions, starting with a global overview of digital technologies in regulatory science. One session was particularly notable: a dialog on the readiness of regulatory science for AI, a focus of this manuscript. Other sessions explored AI, machine learning, and the application of digital technologies in regulated products, public health, and novel applications. Around 200 participants attended the conference, including more than 50 international participants from 15 countries.

**Fig. 1:**
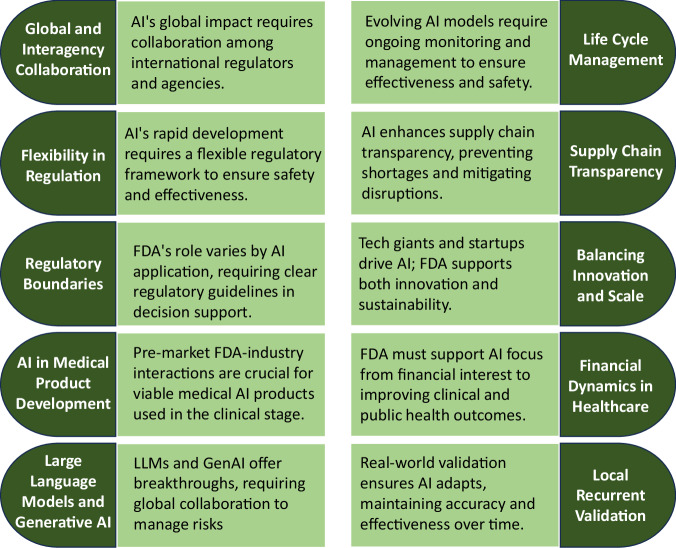
Ten key concepts for AI integration at FDA. Ten key principles guide AI integration in regulatory science, underscoring the FDA’s dual role in regulation and public health and emphasizing the need for adaptable policies to keep pace with rapid AI advancements.

FDA Commissioner Dr. Robert M. Califf kicked off the conference with his welcome remarks focusing on several key points regarding the FDA’s role and challenges in the context of modern technological advancements, particularly AI. He highlighted the significant impact of digital transformation on regulatory science, emphasizing the need for adaptable regulation to keep pace with the rapid advancements in AI. Since AI’s integration into regulatory science is inevitable and already underway, the global regulators must gain a deep understanding to develop adaptable regulatory frameworks. Dr. Califf outlined ten key principles for regulating AI (Fig. 1) and concluded by stressing the FDA’s dual role as a regulatory and public health agency in using AI to enhance patient care and public health^4^.

The event consisted of seven sessions, one of which was a dialog among panelists (most co-authors of the paper), engaging the audience in discussing the question: “Is Regulatory Science Ready for AI?”. This paper presents diverse perspectives from panelists and the audience on building trust in AI for regulatory science. The discussion spanned both technical and societal considerations, emphasizing strategies to maximize AI’s benefits while mitigating potential risks in regulatory applications.

## Opportunities for AI in regulatory science

Given the rapid advancement in emerging technologies across chemical industries and pharmaceutical companies, the global regulatory agencies are increasingly required to conduct safety evaluation and risk assessment of the growing number of chemicals and drugs. AI integration can significantly enhance regulatory efficiency and decision-making^5^. One of the key advantages of AI is its ability to automate information retrieval and synthesis, allowing regulators to conduct systematic reviews more efficiently. This capability adds value to regulatory agencies like the FDA, helping them stay informed in a fast-evolving scientific landscape and promoting more evidence-based regulatory actions.

During the conference, speakers showcased AI tools that were currently used in the regulatory settings. One use case presented AI-driven systematic reviews in toxicology, where AI integrates diverse data sources (e.g., peer-reviewed studies, regulatory databases, and real-world evidence) for improved efficiency and accuracy. In addition, probabilistic modeling was discussed as a method to provide more detailed risk characterizations than traditional binary “safe or unsafe” assessments. With this approach, AI models may evaluate the effects of combined exposures from multiple chemicals with a more realistic picture of cumulative risks. One of the key developments in AI is to deal with multiple modalities to process complex, high-dimensional datasets, which offers an enhanced risk assessment by considering the full spectrum of data types such as omics, genetic, environmental, image, and chemical data. This holistic approach may gain a better understanding of individual and population-level risks, going beyond the evaluation of substances in isolation.

With respect to predictive toxicology, AI models have been developed for predicting toxicological outcomes and drug safety. With the most advanced AI algorithms such as deep learning, AI models can predict the effects of chemicals or drugs more accurately, which may have the added benefit of reducing our reliance on animal use. Moreover, scalability is a benefit of AI in regulatory workflows. Since many regulations follow a stringent and short timeline, AI can process large volumes of data rapidly to identify priority chemicals for further testing or communicate with the sponsors to address potential risk of chemicals early. This type of AI application may address increasing demands while upholding rigorous safety standards. Together, these advancements position AI as a valuable tool for modernizing and scaling regulatory processes.

## Trust is everything for uptake of AI in regulatory science

Besides conducting the annual GSRS conference, GCRSR also establishes technical taskforces to develop a framework for the uptake of specific technologies that are ready to impact global regulatory agencies with a broader application. The GCRSR large language models (LLMs) Taskforce was established in early 2023 to develop a roadmap for the use of LLMs in regulatory application with participations from 13 countries^6^. The team realized that trust is one of the most important aspects of the uptake of LLMs in the regulatory setting. For that, they proposed the TREAT principle, which consists of Trustworthiness, Reproducibility, Explainability, Applicability, and Transparency^7^. While the principle aims to ensure that LLMs are reliable and trustworthy, the discussion explored whether this should serve as a guiding framework for all AI applications in regulatory decision-making. If so, what challenges and implications must be addressed, given that many issues remain unresolved and could hinder practical implementation?

**Fig. 2:**
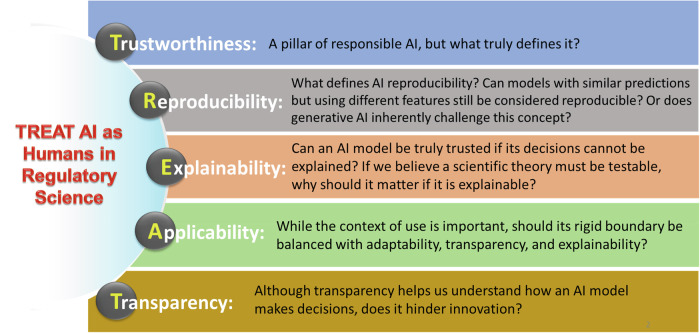
The TREAT principle and the questions for its application in regulatory science. TREAT outlines five key characteristics for AI integration in regulatory science, though their importance varies by application. For instance, can AI models with different features but similar predictions be trusted? If scientific theories must be testable, how critical is explainability? Should a rigid context of use be balanced with adaptability and transparency?

GSRS24 participants agreed that trust is the most important factor for achieving acceptance and integration of AI in regulatory applications. However, there was no consensus on what defines a trustworthy AI, let alone how to measure it. While reproducibility, explainability, applicability, and transparency are commonly cited as essential attributes, their suitability remains a topic of debate. As depicted in Fig. 2, a key question is whether the unique characteristics of AI necessitate a rethink of traditional definitions of these concepts, particularly in guiding the regulatory applications of the rapid advancements in modern AI.

Reproducibility often refers to an experiment or analysis that can be reproduced by others to yield consistent results under identical conditions. Reproducibility serves as a cornerstone of scientific inquiry. It has been argued that the lack of AI reproducibility hinders trust in AI application^8^. However, AI challenges traditional reproducibility norms. The participants pointed out that AI models, particularly machine learning models, could generate distinct yet equally statistically accurate predictions^9^. In this context, the reproducibility was shifted from replicating identical models to maintaining consistent predictive performance. The participants also acknowledged that the very definition of reproducibility could be further complicated by the rise of Generative AI which is designed to generate new information and the new information varies on each run. It was pointed out that these complexities do not negate the importance of reproducibility; rather, new interpretations and understandings of reproducibility tailored to modern AI’s unique characteristics will be required. As AI becomes integral to regulatory science, redefining the meaning and context of reproducibility is necessary to ensure both rigor and relevance in its applications.

Explainability in AI provides insight into the reasoning behind decisions, which is frequently highlighted to foster trust and accountable applications. In domains such as healthcare and regulatory science, explainability has been specifically emphasized for ensuring transparency and mitigating risks. However, some argued that, if an AI model delivered reliable outcomes consistently, the importance of explainability is less significant in application. For example, if people consistently perform reliably, there might be less concerns on explaining their actions. The rationale behind the TREAT principle is to treat AI like humans, where trust is earned through reproducible and consistent performance. Moreover, it was pointed out that in the domain of explaining scientific results, explainability could vary with individual knowledge and training in this field. This explainability’s subjective nature further complicates its objective implementation in regulatory decision-making. It was agreed that explainability represents a balance between trust and performance, requiring careful consideration to meet diverse regulatory science needs.

The applicability domain defines the boundaries where a model’s performance is well defined within specific contexts. The context of use is a key consideration in qualifying biomarkers and drug discovery tools at FDA^10,11^. The question was whether a rigid boundary should be balanced with adaptability, transparency, and continuous monitoring? It was pointed out that applicability may overlook the importance of explainability; even within a defined domain, a ‘black box’ based decision-making process could limit a model’s utility in the fields like regulatory science. A paradigm shift is necessary, prioritizing adaptability and trustworthiness over static applicability. Regulatory science must redefine these principles to align with AI’s evolving capabilities and challenges.

Transparency in AI emphasizes openness about data, design, and decision-making processes. Transparent AI enables trust, accountability, ethical oversight, stakeholder confidence, and promoting collaborative advancements. Some, however, suggested that transparency may not always be practical or essential, as reliable performance may outweigh the need for full disclosure. Most importantly, transparency may hinder innovation, especially in competitive industries. In regulatory science, transparency and innovation should be balanced, where frameworks need to be developed and communicated clearly to improve trust through transparency while accommodating the creative freedom for technological progress.

By ensuring that AI systems are TREAT-compliant, we can enhance user trust and regulatory acceptance of AI technologies. However, while the audience agreed on the general concept of trust in context of the TREAT principle, it was argued that careful consideration be given to dynamic criteria that accommodate the rapid advancement in AI technologies to support regulatory science. Although a consensus was not reached, the dialog furthered our thoughts on AI’s governance and integration across important domains in regulatory science.

## Challenges and considerations

**Fig. 3:**
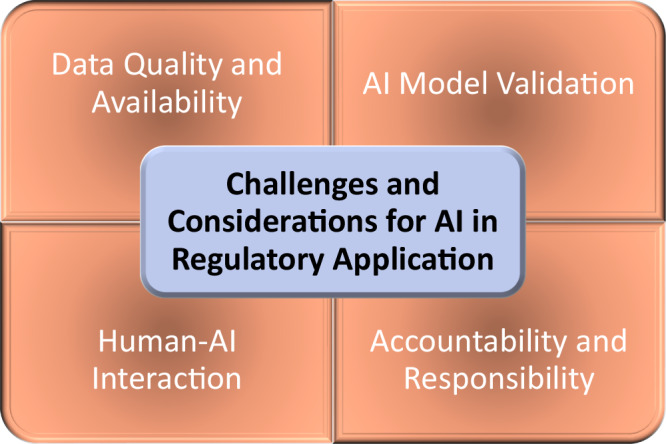
Challenges and considerations for AI integration in regulatory science. Successful AI integration in regulatory science depends on overcoming key challenges related to data quality, model validation, human-AI interaction, and accountability to ensure reliability, transparency, and trust. For data quality and availability, high-quality data is crucial for AI in regulatory science, yet toxicology remains data sparse. Regarding AI model validation, traditional validation methods may not fully assess AI performance in regulatory applications, requiring novel approaches. In terms of human-AI interaction, a “co-pilot” approach, where AI augments human expertise, can build trust along with meaningful oversight to balancing accountability and AI benefits. To achieve accountability and responsibility, clear frameworks are needed to define responsibilities for AI-assisted decisions and establish error-handling guidelines.

While AI offers numerous opportunities, it also presents challenges that require mitigating strategies (Fig. 3). Addressing these challenges will facilitate AI integration into regulatory workflows, including the need for developing competencies and an in-depth understanding of AI. While it is not anticipated that AI will replace human expertise any time soon, using AI to augment decision-making is already a growing reality in regulatory application. This paradigm shift can only be progressed and accelerated through a well-prepared workforce to ensure effective oversight. Proposed strategies include training programs and interdisciplinary collaboration to facilitate a smooth transition to AI-enhanced regulatory frameworks.

### Data quality and availability

AI is a data intensive technology while toxicology is a data-sparse field. To develop a robust model in toxicity assessment, data quality is a prerequisite for a reliable AI application in regulatory science. High-quality and unbiased datasets are crucial, but currently many of them are incomplete. Biases or inaccuracies in training data could lead to serious errors, underscoring the need for standards in data quality for AI-driven regulatory applications. Sharing of data presents another challenge; much of the valuable data on safety of substances reside in proprietary databases or unpublished studies. It was suggested that regulatory bodies such as the FDA may play a role to bring various stakeholders together with mechanisms for secure data-sharing across industry, academia, and regulatory agencies. Some advanced technologies of sharing such as federated learning or blockchain could also facilitate development of AI models in a privacy-preserving fashion.

The other key concern in the adoption of AI for regulatory science is to have an objective way to assess biases embedded in data, particularly in legacy data. For example, the FDA’s Office of Women’s Health was established by congressional mandate in 1994 to advocate for the participation of women in clinical trials and the analyses of data by sex, indicating a poor female representation in the early data. Therefore, it is crucial to include diverse and representative datasets to ensure faithfulness and fairness in AI modeling. Participants stressed the need for equity in regulatory processes to mitigate any bias that could possibly lead to causing harm to vulnerable populations. Addressing these issues is essential to uphold the integrity and inclusivity of regulatory practices.

### AI model validation

Developing robust validation frameworks for AI in regulatory contexts presents unique challenges. Traditional statistical validation methods may not fully capture the performance of complex machine learning models, especially when dealing with high-dimensional data or rare events. Novel approaches to model validation may incorporate uncertainty quantification and sensitivity analysis to ensure that AI systems meet the high standards required for regulatory decision-making. This is specifically important when a model relies on accessing large amounts of low-quality data with the intention of compensating for a lack of high-quality curated datasets. In addition, synthetic data may fill gaps, however, its utility without proper validation^12^ can be problematic.

### Human-AI interaction

The human-AI interaction has been recognized as an important aspect of effectively applying AI in regulatory science. While AI offers efficiency gains, building appropriate trust and validation processes remains key to its success. A gradual, “co-pilot” approach was put forward where AI tools are introduced to augment rather than replace human expertise as comfort and capabilities grow over time. This approach allows for careful validation of AI outputs against human expert judgment, building confidence in the technology and its use for different regulatory applications.

Beyond regulators and decision-makers, the perspective of those affected by regulatory rulings—the *end users* of these regulations—is equally important. Industry stakeholders, healthcare professionals, and the public may have different concerns about the use of AI in the context of TREAT. Ensuring that AI-driven regulatory decisions are TREAT comparable is essential to maintaining public trust and compliance. The concept of *meaningful human oversight* in AI-assisted decision-making must therefore be carefully defined not only for regulatory authorities but also with consideration of those subject to these regulations. Determining the appropriate level of human involvement to ensure both accountability and broad stakeholder confidence while still realizing AI’s benefits remains a key challenge^13^.

### Accountability and responsibility

As AI takes on larger roles in regulatory science, frameworks for accountability must be established that can handle AI’s evolving nature. Such frameworks are essential since regulatory agencies will likely take ultimate responsibility for AI-assisted processes and their outputs. AI is unique and unlike other analytical tools used in regulatory settings; its nature demands that the concept of distributed authority between humans and AI systems be carefully considered. For example, how should responsibility be assessed when errors occur in AI-assisted regulatory decisions? Clear guidelines are needed to delineate the roles and responsibilities of human experts, AI system developers, and the institutions deploying these technologies.

Maintaining public trust is vital for the effective adoption of AI in regulatory science. Transparent communication about AI applications, limitations, and safeguards can foster stakeholder confidence. Engaging the public in open dialogs about the AI use will facilitate the long-term trust and acceptance.

## Future perspectives

An effective starting point for integrating AI in regulatory environments is to focus on areas where efficiency gains can be readily demonstrated with minimal risk. Tasks such as information retrieval and synthesis represent low-hanging fruit, offering clear value while enhancing institutional knowledge and confidence in the technology. Another promising area is the application of AI models, such as AI-facilitated quantitative structure-activity relationships (QSARs), for assessing chemical and drug safety. These models have matured over time, and their appropriate use has been extensively discussed, providing a solid foundation for further exploration.

To implement these applications in regulatory decision-making, establishing “regulatory sandboxes” is crucial. These controlled environments allow for the testing and refinement of AI solutions in various regulatory contexts. Furthermore, developing high-quality reference datasets is essential to establish benchmarks for evaluating AI performance across diverse scenarios. These efforts will enable more robust and reliable integration of AI into regulatory workflows, paving the way for its responsible and impactful use.

International collaboration among global regulators is essential to unlock the potential of AI in the regulatory landscape, addressing both its challenges and opportunities. Such collaboration facilitates capacity-building and enables the effective integration of AI while leveraging data across global agencies. Importantly, it can foster consensus on AI applications that are technically robust, practically relevant, and regulatorily responsible.

A key outcome of this collaboration could be the establishment of inter-agency training programs, a primary goal of GCRSR. Such programs aim to equip regulatory professionals with the technical skills and critical thinking abilities required to oversee AI-driven processes. Additionally, collaborative efforts can support the development of protocols to manage disagreements between AI-generated recommendations and human judgment, ensuring that decisions remain accountable and thoroughly documented. By aligning technical expertise with regulatory frameworks, such initiatives can ensure the responsible and impactful application of AI in global regulatory science.

Transparency and public engagement are essential to gain trust in AI’s role within regulatory science. Proactive communication about AI’s capabilities, limitations, and safeguards can set realistic expectations and demonstrate accountability. Investments in explainable AI tailored to regulatory contexts can bridge the gap between complex models and the need for interpretability. Regular audits and impact assessments further ensure that AI use aligns with ethical standards and regulatory goals.

## Concluding remarks

AI integration into regulatory science is a global issue. GCRSR is playing a role in facilitating its implementation and adoption. AI offers several prominent opportunities to improve the regulatory review process such as manual readings, safety assessment, and institutional knowledge. The GCRSR’s annual GSRS conference serves as a platform to discuss various strategies for AI adoption in regulatory science, such as the TREAT framework as a pathway to achieve efficient, transparent, and trustworthy regulatory processes with AI. Since AI integration in the global regulatory landscape is inevitable, ongoing dialog among stakeholders—facilitated by events like the GSRS—is key to addressing challenges and opportunities, enabling regulatory bodies to make more informed, safe, and timely decisions. It is important to note that the challenges and opportunities for AI integration are dynamic and evolving, requiring flexible and adaptive strategies. A framework for maintaining human oversight, ensuring fairness and transparency, and continuously evaluating the impact of AI-assisted decisions is central to successful AI integration. More importantly, this should be a collaborative effort among global regulators to build a culture of responsible integration, and a commitment to the principles of good regulation, so that AI can be harnessed to meet 21st-century demands in regulatory science.

## Data Availability

No datasets were generated or analyzed during the current study.
